# Implementation of screening tools and brief intervention by health professionals trained by a distance learning course

**DOI:** 10.1186/1940-0640-7-S1-A86

**Published:** 2012-10-09

**Authors:** Maria Lucia O Souza-Formigoni, Ana Paula L Carneiro, Eroy A Silva, Paulina CAV Duarte

**Affiliations:** 1 Department of Psychobiology, Federal University of São Paulo, São Paulo, Brazil; 2 Department of Biological Sciences, Federal University of São Paulo, São Paulo, Brazil; 3 Brazilian National Secretariat for Policies on Drugs (SENAD), Rio de Janeiro, Brazil

## 

There is evidence for the effectiveness of screening and brief intervention (SBI) for risky alcohol and other drug use when administered by trained health professionals. However, there have been no controlled studies on the implementation of BI techniques when health professionals were trained by a distance learning (DL) process. In this study, we evaluated the use of SBI by health professionals trained by DL. All participants were invited by email and regular mail to participate in the project. Those who agreed to participate received, by email, a questionnaire about their use of SBI, including questions on the difficulties associated with implementing SBI, screening tools used (e.g., the Alcohol Use Disorders Identification Test [AUDIT] or the Alcohol, Smoking, and Substance Involvement Screening Test [ASSIST]), and whether or not they conducted BIs. All participants answered a similar questionnaire immediately after the end of the course. Considering both questionnaires, only a few participants had problems conducting BI due to lack of support from their work team. Of the 94% of participants who intended to implement BI in their work, 74% did. After the course, most of the participants approached patients to assess their alcohol (72%) or drug (66%) use and out of those, 64.5% applied the AUDIT and 57.5% applied the ASSIST. Most participants (71%) also felt able to perform BI and to develop strategies to reduce substance use (84%); of these, 83% and 80%, respectively, did it. These results show that health professionals trained by DL can apply SBI. A study to evaluate the effectiveness of BI delivered by DL participants is under way.

